# Diabetes status and cognitive function in middle-aged and older adults in the Canadian longitudinal study on aging

**DOI:** 10.3389/fendo.2023.1293988

**Published:** 2023-12-01

**Authors:** Mohammad Nazmus Sakib, Reza Ramezan, Peter A. Hall

**Affiliations:** ^1^ Department of Health Research Methods, Evidence, and Impact, Faculty of Health Sciences, McMaster University, Hamilton, ON, Canada; ^2^ School of Public Health Sciences, Faculty of Health, University of Waterloo, Waterloo, ON, Canada; ^3^ Department of Statistics and Actuarial Science, University of Waterloo, Waterloo, ON, Canada; ^4^ Centre for Bioengineering and Biotechnology, University of Waterloo, Waterloo, ON, Canada; ^5^ Department of Psychology, University of Waterloo, Waterloo, ON, Canada

**Keywords:** CLSA, cognitive function, executive function, reaction time, memory, diabetes

## Abstract

**Objectives:**

Diabetes is recognized as a significant risk factor for cognitive impairment. However, this association has not been thoroughly examined using large-scale population-based datasets in the Canadian context. The objective of this study was to investigate the potential association between cognitive function and diabetes in a large population-based sample of middle-aged and older Canadians.

**Methods:**

We utilized baseline data from the Canadian Longitudinal Study on Aging (N=30,097) to test our hypotheses, using five indicators of cognitive function (animal fluency, Stroop interference, reaction time, immediate and delayed memory recall). We conducted multivariate multivariable linear regression and subsequently performed tests for moderation analysis with lifestyle factors and health status.

**Results:**

The analysis revealed that type 2 diabetes (T2DM) was associated with lower performance on most cognitive tasks, including those assessing executive function (b=0.60, 95% CI 0.31 to 0.90), reaction time (b=16.94, 95% CI 9.18 to 24.70), immediate memory recall (b=-0.10, 95% CI -0.18 to -0.02), and delayed memory recall (b=-0.12, 95% CI -0.21 to -0.02). However, no significant association was observed between other types of diabetes and cognitive performance. Moderation effects were largely null for T2DM, with the exception of alcohol intake for reaction time, and physical activity for animal fluency.

**Conclusions:**

The study showed that individuals with T2DM exhibit poor performance on tasks that assess executive function, reaction time, and memory. Therefore, optimizing cognitive health among individuals with T2DM should be a priority in primary care. Additionally, further studies should examine this association using longitudinal data.

## Introduction

Type 2 diabetes mellitus (T2DM) is a prevalent chronic condition that affects a significant number of individuals worldwide. Globally, an estimated 462 million people have T2DM, which represents 6.28% of the world’s population (1). In Canada, over 3 million people, or approximately 8.9% of the population, are living with diabetes, making it a notable public health issue (2). People with diabetes are at higher risk of developing macrovascular and microvascular complications, including cardiovascular disease, diabetic retinopathy, neuropathy, and diabetic kidney disease, which can lead to increased mortality, kidney failure, blindness, and reduced quality of life (3).

The association between T2DM and cognitive function is often given only minimal attention in clinical practice, although several previous studies have demonstrated that T2DM is linked to negative cognitive outcomes (4–7). For instance, previous research has shown that T2DM is an independent risk factor associated with a 1.5- to 2.5-fold increased risk of dementia (8, 9). The review of existing literature suggests that T2DM is generally associated with lower cognitive performance (10), particularly in tests of memory, learning, attention, and executive function (11). A recent analysis of the UK Biobank data showed that T2DM is significantly associated with a shorter digit span but not with fluid intelligence, reaction time, visual and prospective memory (12). This indicates that T2DM might predominantly affect higher-order cognitive functions like working memory and executive function. A negative correlation between the degree of cognitive functioning and the number of years since T2DM diagnosis has also been observed (7, 13). The development of cognitive dysfunction is believed to be influenced by microvascular diseases and persistent hyperglycemia that occur in T2DM (11). Additionally, T2DM has been linked to a variety of alterations in the central nervous systems, including cortical atrophy and microstructural anomalies in white matter pathways (11, 14, 15).

It is believed that the association between T2DM and cognitive function is moderated by lifestyle factors and health status. For instance, a higher burden of comorbidities has been shown to be inversely associated with cognitive function (16). According to Wei and colleagues, multimorbidity is linked to both acute decline in cognition and accelerated, persistent cognitive decline over time (17). Physical activity may also moderate the relationship between T2DM and cognitive function. For instance, prior studies have demonstrated that physical activity is not only linked to a reduced risk of cognitive decline (18, 19), but can also improve cognitive function (20). On the other hand, alcohol consumption, especially heavy drinking, has been linked to a higher risk of cognitive dysfunction and dementia (21–23).

Though prior studies have shown a link between T2DM and cognitive performance, there has not been much population-based, large-scale research on this subject in the Canadian context. Furthermore, previous studies have not thoroughly examined the moderating role of lifestyle factors and comorbidity status. Therefore, this study aimed to investigate the relationship between cognitive function and T2DM and determine whether or not lifestyle factors and health status play a role in moderating this relationship. We hypothesize that having diabetes would be associated with decreased performance on cognitive tasks and that lifestyle factors and health status would act as moderators of this association.

## Method

### Data source

Data from the Canadian Longitudinal Study on Aging (CLSA) were used to conduct this analysis. The CLSA is a large-scale, nationwide prospective study in Canada that included 51,338 participants at baseline. The CLSA is made up of two separate cohorts, namely tracking and comprehensive. The tracking cohort consists of 21,241 participants recruited across 10 Canadian provinces. A 60-minute telephone interview was conducted with these subjects. The comprehensive cohort comprises 30,097 participants who were recruited from 25-50 km radius of one of the 11 Data Collection Sites (DCS) (Vancouver, Victoria, Calgary, Winnipeg, Hamilton, Ottawa, Montréal, Sherbrooke, Halifax, and St. John’s) in 7 Canadian provinces (24–26). These participants underwent 90-minute in-person interviews at their homes and visited a data collection facility for a thorough evaluation.

The collection of baseline data for the CLSA began in 2010 and was completed in 2015. Three sources were used to recruit participants for the CLSA: the Canadian Community Health Survey-Healthy Aging (for the CLSA tracking cohort only), provincial health registries, and telephone sampling using random digit dialing (24, 25, 27). The CLSA recruited men and women who were between the ages of 45 to 85 years. However, residents of three Canadian territories, occupants of federally administered First Nations reserves or other First Nations communities in the provinces, Canadian military personnel serving full-time, people residing in long-term care facilities, people who were cognitively impaired at the time of contact, and people who could not communicate in one of the two official languages (English or French) of Canada were excluded during baseline recruitment (24, 25, 27). Each participant provided their written consent to take part in the CLSA. The sampling strategy and study design have been extensively described in other publications (24, 25).

As several of the relevant cognitive variables (such as the Stroop Neurological Screen Test [SNST] and the Choice Reaction Time [CRT] Test) were not available for the tracking cohort participants, this study solely used baseline data of the comprehensive cohort. Therefore, 30,097 Canadian men and women between the ages of 45 and 85 were included in this cross-sectional analysis.

### Measures

#### Dependent variables

##### Stroop neurological screen test

SNST is considered a robust measure of executive function (28, 29). In the task, participants are asked to determine the color of the font used to write a word while ignoring the actual meaning of the word. The time it takes to recognize the color increases in comparison to a baseline condition when the written word is inconsistent with the font color (for example, when “Red” is written in “Green” font).

The CLSA employed the Victoria version of the Stroop task (30). In this task, participants were shown three stimulus cards in succession. The first card was a “neutral” condition where a list of neutral words was printed using various ink colors. Participants were instructed to read the neutral words from left to right for each row. The second card was a “congruent” condition where a number of “X”s were printed with different ink colors. Participants were asked to name the color of the ink used to print each “X”. The third card presented an "incongruent" condition where several color words were printed in a manner such that the color words and the ink color did not coincide (for instance, the word "Green" is written in "Blue" ink). Participants were asked to quickly identify the color of the ink used to write the words, omitting their intended meaning.

The responses of the participants and the time taken to complete each block were recorded. The metric of interest was Stroop interference, which represents the delay in completing the incongruent block due to distracting stimuli compared to the congruent condition. This was calculated by subtracting the time required to finish the congruent block from that of the incongruent block.

##### Choice reaction time test

This task measures general alertness and processing speed (31). The CRT was administered using a touch-enabled computer screen. The task began with four horizontal plus signs and one key sign underneath each plus sign on the screen (30). The plus signs were randomly changed into boxes, and the participants had to press the key sign underneath the box as rapidly as they could. There were 52 iterations of this exercise (30). The computer program automatically generated the test scores. The mean reaction time was calculated by averaging the reaction time of correct responses while omitting timeouts and wrong responses.

##### Animal fluency test

This task is a measure of verbal fluency and is frequently used to screen cognitive impairment and dementia. For this task, participants are asked to recite as many animals as they can in 60 seconds, with one point awarded for each distinct animal correctly named (30). During the CLSA data collection, the responses of the participants were recorded and uploaded to a database. Test scores were then calculated using a validated algorithm (30). In terms of interpretation, it is assumed patient may be in the early stages of dementia or developing cognitive impairment if they can only name 15 animals or fewer in the allotted 60 seconds (32).

##### Rey auditory verbal learning test (Rey I)

This task is a widely used neuropsychological memory test that is highly sensitive in detecting early cognitive decline. In this test, a participant is presented with a list of 15 recorded words and is given 90 seconds to recall as many words as possible, in any order (30, 33). Each correctly recalled word (or approved variant) earns the participant one point, while zero points are awarded for any other word provided. The higher the score, the better the memory and verbal learning performance.

##### Delayed rey auditory verbal learning test (Rey II)

The Rey II is another memory task that requires participants to recall the same list of recorded words played in the Rey I task. In this case, however, they only have 60 seconds to recall as many words as possible in any order (30, 33). The same scoring system applies, with one point being awarded for each primary or variant word correctly recalled, and zero points for any other word provided. By measuring the retention and retrieval of information over a delay period, Rey II adds an additional level of complexity to the assessment.

#### Independent variables

##### Diabetes status

Two variables were used to determine diabetes status (and type). Participants were questioned about their diabetes status as follows: “Has a doctor ever told you that you have diabetes, borderline diabetes or that your blood sugar is high?” (34). Those who responded “No” were assumed not to have diabetes. The following question was then asked to participants who responded “Yes” in order to determine the type of diabetes: “Were you diagnosed with: Type I, Type II, or Neither” (34). A nominal variable was then created using the following categories: Type II diabetes, other types of diabetes, and no diabetes.

### Covariates and moderators

#### Age

The age of the participants (in years) was determined based on their birthdate and provided as a numerical variable in the data set (35). For descriptive analyses, it was further transformed into an ordinal variable with the following age ranges: 45–54, 55–64, 65–74, and 75+ years.

*Sex.* Participants were asked to report their biological sex at birth; men were given the code 1, and women were given the code 0 (35).

#### Ethnicity

This variable was coded as 1 and 0, where 1 represents White ethnicity, and 0 represents ethnicities other than White (35).

#### Income

Income was evaluated based on the overall household income. The following question was asked to the participants: “What is your best estimate of the total household income received by all household members, from all sources, before taxes and deductions, in the past 12 months?” (35). The following ranges were used to categorize this variable:< $20,000, $20,000–$50,000, $50,000–$100,000, $100,000–$150,000, and ≥$150,000 (35). The missing values for this variable (*n* = 1393) were recoded as “no response.”

#### Education

Education level was determined using two variables. Participants were first asked if they had completed their high school education. Next, participants were asked: “Have you received any other education that could be counted toward a degree, certificate, or diploma from an educational institution?” (35). Respondents who answered “no” were regarded as having a level of education that was “high school or below”. The following question was then posed to those who responded “yes” in order to determine the level of education attained: “What is the highest degree, certificate, or diploma you have obtained?” (35). These variables were combined to create an ordinal variable that had three categories: “high school or less”, “certificates or degrees below a bachelor’s”, and “bachelor’s or higher”.

#### Residence

Residential area was classified as either rural or urban. In the original variable, the residence was classified into the following categories: rural, urban core, urban fringe, urban population center outside a census metropolitan area, and census agglomeration, secondary core, and postal code link to dissemination area (35). This variable was recoded as rural and urban, with urban including all nonrural groups.

#### Body mass index

The dataset included each participant’s height (in metres) and weight (in kilograms) (34). The formula for the BMI variable was weight in kg divided by height in m^2^. A BMI categorical variable was also created, with the following ranges: underweight (BMI< 18.5), normal (BMI = 18.5-24.9), overweight (BMI = 25–29.9), and obese (BMI 30).

#### Physical activity

The following question was asked to the participants: “Over the past 7 days, how often did you take a walk outside your home or yard for any reason? For example, for pleasure or exercise, walking to work, walking the dog, etc.” (36). The following responses options were provided: never, seldom (1 to 2 days), sometimes (3 to 4 days) and often (5 to 7 days). This variable was dichotomized as follows: seldom ( ≤ 2 days) and often (>3 days).

#### Comorbidity

The sum of the following 25 chronic conditions was used to create a comorbidity index variable: chronic obstructive pulmonary disease, asthma, heart disease, heart attack, hypertension, stroke, peripheral vascular disease, Parkinson’s disease, epilepsy, multiple sclerosis, dementia, migraine, rheumatoid arthritis, osteoarthritis, other arthritis, back problems, hyperthyroidism, hypothyroidism, depression, mood disorder, anxiety disorder, cancer, bowel disorder, stomach ulcer, and kidney disease (34, 37). An ordinal comorbidity variable was also created as follows with the following categories: no comorbidity, 1-2 comorbidities and > 2 comorbidities.

#### Alcohol intake

To understand the level of alcohol consumption, participants were asked the following question: “About how often during the past 12 months did you drink alcohol?” (35). The response options were almost every day (including 6 times a week), 4-5 times a week, 2-3 times a week, once a week, 2-3 times a month, about once a month, less than once a month, and never. This variable was later dichotomized into two categories: ≤ 1 time per week and 2-7 times per week.

### Statistical analyses

The R statistical software package, version 4.0.5, was used for statistical analysis. In accordance with the recommendation of the CLSA, we employed trimmed weights for descriptive analyses and analytic weights for inferential analyses (27). The study variables were first subjected to descriptive analysis. Weighted percentages and means were calculated for each of the categorical and continuous variables, respectively.

Multivariate multivariable regressions were conducted to assess the relationship between cognitive function and diabetes status. A total of 3 models were evaluated. Model 1 investigated the relationship between cognitive performance and diabetes status in an unadjusted model. Model 2 was a partially adjusted model controlling for sociodemographic variables (e.g., age, sex, ethnicity, income and education). Model 3 was a fully adjusted model further controlling for residence, physical activity, alcohol consumption, and comorbidity.

Finally, moderation analyses were performed to determine if the relationship between T2DM status and cognitive outcomes varied as a function of a number of exogenous variables. Physical activity, alcohol consumption, and comorbidity load were all examined as putative moderators. In all analyses, two-sided tests with 95% confidence intervals were used, and a significance level of *P*<.05 was applied to determine statistical significance.

## Results

**Table 1** provides an overview of the sociodemographic, lifestyle, and health-related characteristics of the CLSA comprehensive cohort (*n* = 30,097). The participants were a mean age of 59.49 years and 50.36% were female. The majority of participants identified as Caucasian (94.7%), and a large proportion reported an annual household income of $50,000 or more (72.29%) and at least a bachelor’s degree (46.43%). Most participants resided in urban areas (91.53%). The mean number of comorbidities was 2.35 (95% CI 2.32, 2.38) but 42.97% of participants reported having at least 1-2 comorbidities. The average BMI of the participants was 27.8 (95% CI 27.72, 27.88), and 27.47% were obese. In terms of lifestyle behaviors, the majority reported frequent walking activities (68.5%) and infrequent drinking ( ≤ 1 time per week, 50.53%). The majority of participants did not have diabetes (84.73%); 7.45% had a diagnosis of T2DM and 7.82% had a diagnosis of “T1DM or other type of diabetes.

**Table 1:** Sample characteristics.

Variables	Weighted percentage/mean (95% CI)
**Age**	59.49 (59.35, 59.63)
**Animal fluency**	20.33 (20.25, 20.41)
**Stroop interference**	9.95 (9.86, 10.05)
**Reaction time**	797.29 (794.89, 799.68)
**Rey I**	6.04 (6.01, 6.07)
**Rey II**	4.30 (4.27, 4.33)
**BMI**	27.80 (27.72, 27.88)
**Comorbidity**	2.35 (2.32, 2.38)
Sex	
Male	49.64 (48.92, 50.35)
Female	50.36 (49.65, 51.08)
Ethnicity	
Non-white	5.30 (4.96, 5.65)
White	94.70 (94.35, 95.04)
Income	
No response	5.60 (5.29, 5.91)
< $20,000	4.42 (4.17, 4.68)
$20,000 to< $50,000	17.69 (17.20, 18.19)
$50,000 to< $100,000	31.43 (30.77, 32.08)
$100,000 to< $150,000	20.94 (20.33, 21.55)
< $150,000 or more	19.92 (19.31, 20.53)
Education	
High school or less	13.85 (13.38, 14.32)
Below bachelor	39.73 (39.03, 40.43)
Bachelor or above	46.43 (45.72, 47.14)
Residence	
Urban	91.53 (91.14, 91.93)
Rural	8.47 (8.07, 8.86)
Physical Activity	
Seldom	31.45 (30.79, 32.10)
Often	68.55 (67.90, 69.21)
Comorbidity	
None	17.92 (17.33, 18.50)
1-2	42.97 (42.26, 43.69)
> 2	39.11 (38.42, 39.79)
Alcohol intake	
≤ 1 time per week	50.53 (49.80, 51.25)
2-7 times per week	49.47 (48.75, 50.20)
BMI	
Normal	31.85 (31.18, 32.52)
Underweight	0.70 (0.59, 0.82)
Overweight	39.98 (39.27, 40.68)
Obese	27.47 (26.84, 28.10)
Diabetes	
None	84.73 (84.24, 85.22)
Other type	7.82 (7.45, 8.19)
Type 2 diabetes	7.45 (7.11, 7.79)

In unadjusted models, T2DM was associated with significantly worse performance on all cognitive outcomes (**Table 2**). In fully adjusted models, most of the sociodemographic variables were significantly associated with cognitive function. When compared to women, men exhibited better performance in the reaction time task (b = -19.74, 95% CI -23.75, -15.72, p< 0.001) but worse performance in the Stroop task (b = 0.47, 95% CI 0.32, 0.62, p< 0.001) and the memory task (Rey I: b = -0.84, 95% CI -0.88, -0.80, p< 0.001; Rey II: b = -1.01, 95% CI -1.06, -0.96, p< 0.001). BMI was not significantly associated with most of the cognitive outcomes, except for the Stroop task, where an increase in BMI was associated with higher Stroop interference (b = 0.02, 95% CI 0.00, 0.03; p = 0.018). Having two or more comorbidities showed a significant association with animal fluency and reaction time tasks, but the direction of the association suggests better verbal fluency (b = 0.20, 95% CI 0.02, 0.39, p = 0.033) and slower reaction time (b = 6.09, 95% CI 0.20, 11.99, p = 0.043). **Appendix Table 1** presents the associations between cognitive variables and other sociodemographic variables.

**Table 2:** Multivariate multivariable regression with cognitive function as an outcome variable (Model 1-3).

Variables	Animal fluency		Stroop interference		Mean reaction		Rey I (immediate)		Rey II (delayed)	
	*b* (95% CI)	*p*	*b* (95% CI)	*p*	*b* (95% CI)	*p*	*b* (95% CI)	*p*	*b* (95% CI)	*p*
	Model 1 (unadjusted)									
**No diabetes**	Ref		Ref		Ref		Ref		Ref	
**Type 2 Diabetes**	**-1.67** **(-1.93, -1.41)**	**<0.001**	**2.15** **(1.85, 2.45)**	**<0.001**	**49.58** **(41.63, 57.53)**	**<0.001**	**-0.64** **(-0.73, -0.56)**	**<0.001**	**-0.73** **(-0.83, -0.63)**	**<0.001**
**Other Type**	**-0.64** **(-0.89, -0.38)**	**<0.001**	**0.62** **(0.33, 0.91)**	**<0.001**	**17.37** **(9.63, 25.11)**	**<0.001**	**-0.19** **(-0.27, -0.11)**	**<0.001**	**-0.21** **(-0.30, -0.11)**	**<0.001**
	Model 2 (demographic adjusted)									
**No diabetes**	Ref		Ref		Ref		Ref		Ref	
**Type 2 Diabetes**	-0.21 (-0.45, 0.03)	0.092	**0.78** **(0.49, 1.06)**	**<0.001**	**16.48** **(8.95, 4.02)**	**<0.001**	**-0.13** **(-0.21, - 0.05)**	**0.001**	**-0.16** **(-0.25, -0.07)**	**0.001**
**Other Type**	0.02 (-0.21, 0.25)	0.839	0.02 (-0.25, 0.30)	0.870	3.18 (-4.06, 0.42)	0.390	0.03 (-0.05, 0.10)	0.481	0.03 (-0.06, 0.12)	0.520
	Model 3 (fully adjusted)									
**No diabetes**	Ref		Ref		Ref		Ref		Ref	
**Type 2 Diabetes**	-0.14 (-0.39, 0.10)	0.252	**0.60** **(0.31, 0.90)**	**<0.001**	**16.94** **(9.18, 24.70)**	**<0.001**	**-0.10** **(-0.18, -0.02)**	**0.018**	**-0.12** **(-0.21, -0.02)**	**0.014**
**Other Type**	0.02 (-0.21, 0.25)	0.870	-0.07 (-0.34, 0.21)	0.642	3.28 (-4.05, 0.60)	0.380	0.04 (-0.04, 0.12)	0.313	0.05 (-0.04, 0.14)	0.291

Model 1 is unadjusted model. Model 2 is adjusted for sociodemographic variables, such as age, sex, ethnicity, income and education. Model 3 is further adjusted for residence, BMI, comorbidity, physical activity, and alcohol intake.

Bold values indicate statistically significant findings.

Consistent with our hypotheses, there was a reliable association between T2DM and lower Stroop performance (b = 0.60, 95% CI 0.31, 0.90, p< 0.00), slower reaction time (b = 16.94, 95% CI 9.18, 24.70, p< 0.001), lower performance on the Rey I (b = -0.10, 95% CI -0.18, -0.02, p = 0.018) and Rey II (b = -0.12, 95% CI -0.21, -0.02, p = 0.014) memory test in fully adjusted models. Most moderating effects were null. However, T2DM effects on simple reaction time were amplified for those who consumed alcohol 2-7 times per week (b = -16.19, 95% CI -32.32, -0.07, p = 0.049), and animal fluency effects were amplified for those who “seldom” engaged in physical activity (b = 0.64, 95% CI 0.16, 1.13, p = 0.009) (**Table 3**). In a separate analysis, we examined the categorical BMI variable in interaction with the diabetes status. The results showed that obesity status was significantly associated with animal fluency (b = -0.21, 95% CI -0.39, -0.03, p = 0.023), Stroop interference (b = 0.32, 95% CI 0.11, 0.54, p = 0.004), and Rey I (b = -0.08, 95% CI -0.14, -0.02, p = 0.011) and Rey II (b = -0.12, 95% CI -0.19, -0.05, p = 0.001) memory tasks. However, the interaction terms were mostly nonsignificant (**Appendix Table 2**).

**Table 3:** Moderation analyses (Model 4-6).

Variables	Animal fluency		Stroop interference		Mean reaction		Rey I (immediate)		Rey II (delayed)	
	*b* (95% CI)	*p*	*b* (95% CI)	*p*	*b* (95% CI)	*p*	*b* (95% CI)	*p*	*b* (95% CI)	*p*
	Model 4 (interaction with comorbidity status)									
Diabetes status										
**No diabetes**	Ref		Ref		Ref		Ref		Ref	
**Type 2 Diabetes**	-0.41 (-1.29, 0.48)	0.365	0.34 (-0.71, 1.38)	0.525	9.18 (-18.56, 36.92)	0.517	0.04 (-0.25, 0.33)	0.802	-0.03 (-0.36, 0.31)	0.868
**Other diabetes**	**-0.80** **(-1.51, -0.09)**	**0.028**	0.00 (-0.84, 0.85)	0.993	-0.04 (-22.36, 22.28)	0.997	0.20 (-0.04, 0.43)	0.097	0.13 (-0.14, 0.40)	0.359
Comorbidity status										
**No Comorbidity**	Ref		Ref		Ref		Ref		Ref	
**1-2 Comorbidities**	-0.01 (-0.20, 0.17)	0.890	0.12 (-0.10, 0.34)	0.284	2.69 (-3.08, 8.45)	0.361	-0.01 (-0.07, 0.05)	0.800	0.01 (-0.06, 0.08)	0.684
**>2 comorbidities**	0.14 (-0.06, 0.34)	0.164	0.16 (-0.07, 0.39)	0.178	4.85 (-1.35, 11.04)	0.125	0.02 (-0.04, 0.09)	0.520	0.01 (-0.06, 0.09)	0.745
Interaction										
T2DM*1-2 Comorbidities	0.34 (-0.63, 1.31)	0.495	0.01 (-1.14, 1.16)	0.987	2.68 (-27.71, 33.07)	0.863	-0.09 (-0.41, 0.23)	0.573	-0.02 (-0.39, 0.35)	0.921
T2DM*>2 comorbidities	0.79 (-0.01, 1.60)	0.053	-0.21 (-1.16, 0.75)	0.671	-0.55 (-25.79, 24.69)	0.966	-0.20 (-0.46, 0.07)	0.140	-0.05 (-0.35, 0.26)	0.758
Other diabetes*1-2 Comorbidities	0.27 (-0.66, 1.21)	0.569	0.46 (-0.64, 1.57)	0.411	12.19 (-17.21, 41.58)	0.416	-0.18 (-0.49, 0.13)	0.246	-0.15 (-0.50, 0.21)	0.412
Other diabetes*>2 comorbidities	**1.00** **(0.22, 1.78)**	**0.012**	0.03 (-0.89, 0.95)	0.950	7.11 (-17.39, 31.62)	0.569	-0.16 (-0.42, 0.09)	0.210	-0.12 (-0.42, 0.18)	0.425
	Model 5 (interaction with alcohol intake)									
Diabetes status										
**No diabetes**	Ref		Ref		Ref		Ref		Ref	
**Type 2 Diabetes**	-0.11 (-0.40, 0.19)	0.485	**0.77** **(0.42, 1.12)**	**<0.001**	**22.24** **(12.95, 1.52)**	**<0.001**	**-0.12** **(-0.21, -0.02)**	**0.020**	**-0.15** **(-0.26, -0.03)**	**0.010**
**Other Type**	0.16 (-0.15, 0.47)	0.312	-0.15 (-0.52, 0.21)	0.416	8.39 (-1.28, 18.06)	0.089	0.05 (-0.05, 0.15)	0.317	0.05 (-0.06, 0.17)	0.362
Alcohol intake										
** ≤ 1 time weekly**	Ref		Ref		Ref		Ref		Ref	
**2-7 times weekly**	**0.36** **(0.23, 0.50)**	**<0.001**	**-0.39** **(-0.56, -0.23)**	**<0.001**	1.27 (-3.10, 5.64)	0.569	**0.09** **(0.05, 0.14)**	**<0.001**	**0.09** **(0.04, 0.15)**	**0.001**
Interaction										
T2DM*2-7 times weekly	-0.11 (-0.62, 0.41)	0.685	-0.52 (-1.13, 0.08)	0.092	**-16.19** **(-32.32, -0.07)**	**0.049**	0.05 (-0.11, 0.22)	0.526	0.09 (-0.10, 0.29)	0.355
Other Diabetes*2-7 times weekly	-0.32 (-0.79, 0.14)	0.176	0.21 (-0.34, 0.76)	0.459	-11.56 (-26.15, 3.04)	0.121	-0.03 (-0.18, 0.12)	0.708	-0.02 (-0.19, 0.16)	0.849
	Model 6 (interaction with physical activity)									
Diabetes status										
**No diabetes**	Ref		Ref		Ref					
**Type 2 Diabetes**	**-0.40** **(-0.72, -0.09)**	**0.012**	**0.67** **(0.30, 1.04)**	**<0.001**	**17.88** **(8.02, 27.75)**	**<0.001**	-0.09 (-0.19, 0.02)	0.094	**-0.12** **(-0.24, 0.00)**	**0.047**
**Other Type**	-0.08 (-0.36, 0.21)	0.597	0.07 (-0.27, 0.40)	0.691	9.47 (0.56, 18.37)	0.037	0.02 (-0.07, 0.12)	0.624	0.00 (-0.11, 0.10)	0.962
Physical activity (Walking)										
**Frequent**	Ref		Ref		Ref		Ref		Ref	
**Seldom**	**-0.63** **(-0.78, -0.48)**	**<0.001**	0.06 (-0.11, 0.23)	0.504	1.33 (-3.25, 5.92)	0.569	**-0.06** **(-0.11, -0.01)**	**0.013**	**-0.08** **(-0.14, -0.03)**	**0.003**
Interaction										
T2DM*Seldom	**0.64** **(0.16, 1.13)**	**0.009**	-0.17 (-0.74, 0.40)	0.550	-2.87 (-18.01, 12.27)	0.710	-0.02 (-0.18, 0.14)	0.772	0.01 (-0.17, 0.19)	0.899
Other diabetes*Seldom	0.30 (-0.19, 0.78)	0.235	-0.41 (-0.98, 0.17)	0.169	**-18.76** **(-34.09, -3.43)**	**0.016**	0.05 (-0.11, 0.21)	0.553	0.15 (-0.03, 0.34)	0.108

Bold values indicate statistically significant findings.

## Discussion

Using baseline data from that Canadian Longitudinal Study on Aging (CLSA), we examined the association between diabetes status and several domains of cognitive function, including executive function, simple processing speed, and memory. Our findings indicated that T2DM was associated with impaired cognitive task performance compared to those without diabetes on all five cognitive indicators, with substantial effects on all tests in unadjusted models, and across most tests in fully adjusted models. Other forms of diabetes were not associated with significantly impaired performance on any of the cognitive indicators in fully adjusted models. Our findings are in line with previous literature, including a prior meta-analysis documenting reliable deficits in executive function among those living with T2DM (38, 39). Moderation analyses suggest that frequent alcohol usage and sedentary behavior modify some of the cognitive impact of T2DM. These moderation effects may be a function of greater range of variability in each of these. Given that most moderation effects were null, the most prudent interpretation is that T2DM status is associated with cognitive outcomes with significant uniformity across lifestyle and comorbidity levels.

The current findings are important to the extent that they document reliable differences in cognitive measures among those living with T2DM. The executive functions and simple reaction time are both important in everyday life domains. For example, the ability to think quickly through problems and solve them effectively, and to react to real-time changes in the physical environment may be hindered by slower cognitive processing speed (i.e., the cognitive ability underlying simple reaction time task performance). Likewise, a large volume of research links executive function task performance with impaired regulation of reflexive behaviors, thoughts and emotional responses over time. The ability to keep in check reflexive behaviors and emotional responses is important for many domains of interpersonal functioning in the home, workplace, and social spheres. The provision of social support from others, for instance, can be thwarted by such tendencies, leaving someone with diabetes both adversely affected on a cognitive level, but also vulnerable on a social level, in terms of available emotional supports. Protecting and optimizing brain health may be important for those living with T2DM, in part because such sensitive and consequential cognitive functions are likely to be affected. Among known protective factors, exercise and avoidance of alcohol and other substances of abuse may be important factors for protecting cognitive health in the T2DM context, above and beyond simply facilitating the metabolic aspects of disease management. Given that effects were stronger for those with more alcohol consumption and more sedentary lifestyles, these should be discouraged among those with T2DM, using brain health as a rationale.

Form the above perspective, if reliable decrements in processing speed and executive function are an outcome of T2DM disease process itself, it is possible that good glycemic control may be a mitigating factor. This possibility should be explored in future analyses involving CLSA and similar datasets. Some prior studies have shown that favorable glycemic control is associated with better performance on cognitive tasks in other studies (40–42), although it is not clear if this reflects a cause or consequence of the glycemic control.

The exploration of the underlying mechanisms of T2DM-related cognitive decline is beyond the scope of this paper. However, it is important to recognize that cognitive impairment in T2DM can result from various pathological processes. Commonly hypothesized pathways include small-vessel disease and stroke, chronic hyperglycemia, severe hypoglycemic episodes, insulin resistance and hyperinsulinemia, and chronic inflammation (43). Patients with T2DM frequently present with multiple cardiovascular risk factors, including hypertension, obesity, dyslipidemia, hyperinsulinemia, and proinflammatory states. It is believed that the convergence of these major risk factors accelerates small vessel disease and stroke, eventually leading to the development of cognitive impairment and dementia (44, 45). Prior research has consistently demonstrated a reliable association between chronic hyperglycemia and diminished cognitive performance in patients with T2DM (9, 46, 47). Notably, postprandial hyperglycemia exhibits a stronger association with cognitive impairment when compared to fasting blood glucose (48, 49). Conversely, maintaining tight glycemic control can decelerate cognitive decline, as indicated in a recent meta-analysis by Tang and colleagues (42). Hyperglycemia can lead to cerebral microvascular alterations, irregularities in synaptic plasticity, increased oxidative stress, and the accumulation of advanced glycation end products, all of which contribute to the development of cognitive impairment (50, 51).

While optimal glycemic control is desired for T2DM patients, it is important to note that severe hypoglycemia can often occur in the pursuit of intensive glucose control. Severe hypoglycemia may lead to neuronal cell death, platelet aggregation, and fibrinogen formation, which can result in permanent neurological damage and accelerate cognitive impairment (52). Previous reports suggest that recurrent incidents of severe hypoglycemia can increase the risk of cognitive impairment by a factor of 1.5 to 2.0 (52, 53). Individuals with T2DM typically exhibit insulin resistance and compensatory hyperinsulinemia, both of which play crucial roles in mediating cognitive impairment. Insulin possesses vital neurotropic properties, with its receptors being abundant in brain regions associated with learning and memory (54). Prolonged hyperinsulinemia is known to downregulate insulin receptors, resulting in impaired insulin transport into brain tissues (55) and contributing to learning and memory deficits, possibly through neuroglial energy crises (54, 56). Additionally, hyperinsulinemia can disrupt the metabolism of amyloid beta protein, leading to its accumulation and toxic effects in the brain (50).

Patients with T2DM often present higher levels of circulating inflammatory markers (57). Evidence from previous studies suggests that elevated levels of inflammatory cytokines may be linked to diminished cognitive abilities in T2DM patients (9, 46). Increased cytokine levels are believed to contribute to dementia either directly affecting the brain or by fostering vascular disease or insulin resistance (58). Dysregulation of the hypothalamic-pituitary axis and high cortisol levels in diabetic patients are also thought to contribute to cognitive impairment by facilitating microvascular abnormalities (9, 46). In terms of genetic predisposition, earlier studies have reported that individuals with both diabetes and an APOE ϵ4 allele exhibit more pronounced cortical changes, reductions in cognitive function, and an increased risk for dementia (59–61).

There are a number of important strengths and limitations of the current study. In terms of the former, the CLSA is a very large and demographically diverse population cohort, with ample statistical power to detect subtle effects. Likewise, all of the indicators of cognitive function were standardized and widely used measures of the underlying cognitive constructs. Limitations include the use of self-reported diabetes status in the CLSA as well as sub-typing into Type 1, Type 2 or other. Recall biases and lack of knowledge about their own status may impact the validity of this measure of diabetes status. Further, it is estimated that those with T2DM have likely had the disease for several years prior to formal diagnosis, meaning that a number of those classified as diabetic in this (and any) sample is likely a subset of the true number; the existence of false negatives may dilute the strength of relationship between diabetes status and cognitive consequences of diabetes. From this perspective, the associations between T2DM and cognitive function may be underestimated. Lastly, it is important to note that given the predominant representation of Caucasian participants residing in urban areas within the CLSA comprehensive cohort, the findings of this study may not be entirely representative of a more diverse or wider population.

## Conclusion

In conclusion, we found evidence that the presence of T2DM was associated with worse performance on tests of simple processing speed, executive function and memory in a large sample of middle-aged and older adults. Findings were robust to adjustment for a wide variety of demographic and disease-related confounders. The moderation analyses were mostly nonsignificant, indicating largely uniform decrements across lifestyle and comorbidity categories. Future studies should explore prospective relationships between T2DM and cognitive outcomes over longer periods of time, using functional, structural and connectivity analyses.

## Data availability statement

The data analyzed in this study is subject to the following licenses/restrictions: Data are available from the Canadian Longitudinal Study on Aging (www.clsa-elcv.ca) for researchers who meet the criteria for access to de-identified CLSA data. Requests to access these datasets should be directed to https://www.clsa-elcv.ca/data-access.

## Ethics statement

The studies involving humans were reviewed and approved by the Office of Research Ethics at the University of Waterloo. The patients/participants in the CLSA provided their written informed consent to participate in this study.

## Author contributions

MNS: Conceptualization, Data curation, Formal Analysis, Investigation, Methodology, Project administration, Resources, Software, Validation, Writing – original draft, Writing – review & editing. RR: Formal Analysis, Software, Writing - review & editing. PH: Conceptualization, Funding acquisition, Investigation, Methodology, Project administration, Resources, Supervision, Validation, Writing – review & editing.

## Appendix

**Table 1:** Fully adjusted model (Model 3) for the association between diabetes and cognitive function.

Variables	Animal fluency		Stroop interference		Mean reaction		Rey I (immediate)		Rey II (delayed)	
	*b* (95% CI)	*p*	*b* (95% CI)	*p*	*b* (95% CI)	*p*	*b* (95% CI)	*p*	*b* (95% CI)	*p*
**Age**	**-0.16** **(-0.16, -0.15)**	**<0.001**	**0.19** **(0.18, 0.20)**	**<0.001**	**5.75** **(5.53, 5.97)**	**<0.001**	**-0.05** **(-0.05, -0.05)**	**<0.001**	**-0.06** **(-0.06, -0.06)**	**<0.001**
Sex										
Female	Ref		Ref		Ref		Ref		Ref	
Male	0.09 (-0.03, 0.22)	0.152	**0.47** **(0.32, 0.62)**	**<0.001**	**-19.74** **(-23.75, -15.72)**	**<0.001**	**-0.84** **(-0.88, -0.80)**	**<0.001**	**-1.01** **(-1.06, -0.96)**	**<0.001**
Ethnicity										
Non-white	Ref		Ref		Ref		Ref		Ref	
White	**3.39** **(3.09, 3.70)**	**<0.001**	**-1.04** **(-1.40, -0.68)**	**<0.001**	**-74.10** **(-83.70, -64.49)**	**<0.001**	**0.45** **(0.35, 0.55)**	**<0.001**	**0.50** **(0.38, 0.61)**	**<0.001**
Income										
No response	**-1.16** **(-1.48, -0.84)**	**<0.001**	0.35 (-0.02, 0.73)	0.067	**10.30** **(0.34, 20.26)**	**0.043**	**-0.26** **(-0.37, -0.16)**	**<0.001**	**-0.29** **(-0.41, -0.17)**	**<0.001**
< $20,000	**-1.88** **(-2.25, -1.51)**	**<0.001**	**1.99** **(1.56, 2.42)**	**<0.001**	**46.39** **(34.89, 57.88)**	**<0.001**	**-0.73** **(-0.85, -0.61)**	**<0.001**	**-0.44** **(-0.58, -0.30)**	**<0.001**
$20,000 to< $50,000	**-1.31** **(-1.54, -1.08)**	**<0.001**	**0.93** **(0.66, 1.20)**	**<0.001**	**22.83** **(15.65, 30.01)**	**<0.001**	**-0.37** **(-0.45, -0.30)**	**<0.001**	**-0.14** **(-0.23, -0.06)**	**0.001**
$50,000 to< $100,000	**-0.76** **(-0.94, -0.58)**	**<0.001**	0.09 (-0.12, 0.31)	0.392	**9.73** **(3.98, 15.48)**	**0.001**	**-0.14** **(-0.20, -0.08)**	**<0.001**	0.03 (-0.04, 0.10)	0.362
$100,000 to< $150,000	**-0.25** **(-0.44, -0.06)**	**0.009**	0.07 (-0.15, 0.30)	0.513	**6.54** **(0.59, 12.49)**	**0.031**	-0.06 (-0.12, 0.00)	0.065	**0.11** **(0.03, 0.18)**	**0.004**
< $150,000 or more	Ref		Ref		Ref		Ref		Ref	
Education										
High school or less	**-2.79** **(-3.00, -2.58)**	**<0.001**	**1.53** **(1.29, 1.78)**	**<0.001**	**7.78** **(1.31, 14.25)**	**0.018**	**-0.86** **(-0.93, -0.79)**	**<0.001**	**-0.90** **(-0.98, -0.83)**	**<0.001**
Below bachelor	**-1.80** **(-1.94, -1.66)**	**<0.001**	**0.74** **(0.57, 0.90)**	**<0.001**	**4.98** **(0.61, 9.34)**	**0.025**	**-0.51** **(-0.56, -0.47)**	**<0.001**	**-0.55** **(-0.60, -0.49)**	**<0.001**
Bachelor or above	Ref		Ref		Ref		Ref		Ref	
Residence										
Urban	Ref		Ref		Ref		Ref		Ref	
Rural	-0.21 (-0.43, 0.01)	0.065	0.23 (-0.03, 0.49)	0.086	**-15.88** **(-22.77, -8.99)**	**<0.001**	0.05 (-0.02, 0.13)	0.149	-0.03 (-0.11, 0.06)	0.515
**BMI**	0.00 (-0.01, 0.01)	0.973	**0.02** **(0.00, 0.03)**	**0.018**	-0.32 (-0.70, 0.06)	0.095	0.00 (-0.01, 0.00)	0.215	0.00 (-0.01, 0.00)	0.153
Comorbidity										
None	Ref		Ref		Ref		Ref		Ref	
1-2	0.03 (-0.14, 0.21)	0.712	0.10 (-0.11, 0.31)	0.353	2.49 (-3.03, 8.00)	0.377	-0.02 (-0.08, 0.04)	0.526	0.01 (-0.05, 0.08)	0.663
> 2	**0.20** **(0.02, 0.39)**	**0.033**	0.20 (-0.03, 0.42)	0.082	**6.09** **(0.20, 11.99)**	**0.043**	0.00 (-0.06, 0.07)	0.876	0.00 (-0.08, 0.07)	0.903
Alcohol intake										
** ≤ ** 1 time per week	Ref		Ref		Ref		Ref		Ref	
2-7 times per week	**0.33** **(0.20, 0.46)**	**<0.001**	**-0.41** **(-0.56, -0.25)**	**<0.001**	-0.59 (-4.68,3.50)	0.776	**0.10** **(0.05, 0.14)**	**<0.001**	**0.10** **(0.05, 0.15)**	**<0.001**
Physical Activity										
Seldom	**-0.55** **(-0.69, -0.42)**	**<0.001**	0.01 (-0.15, 0.17)	0.881	-0.40 (-4.61, 3.80)	0.851	**-0.06** **(-0.10, -0.02)**	**0.009**	**-0.07** **(-0.12, -0.02)**	**0.006**
Often	Ref		Ref		Ref		Ref		Ref	
Diabetes										
No diabetes	Ref		Ref		Ref		Ref		Ref	
Type 2 diabetes	-0.14 (-0.39, 0.10)	0.252	**0.60** **(0.31, 0.90)**	**<0.001**	**16.94** **(9.18, 24.70)**	**<0.001**	**-0.10** **(-0.18, -0.02)**	**0.018**	**-0.12** **(-0.21, -0.02)**	**0.014**
Other type	0.02 (-0.21, 0.25)	0.870	-0.07 (-0.34, 0.21)	0.642	3.28 (-4.05, 0.60)	0.380	0.04 (-0.04, 0.12)	0.313	0.05 (-0.04, 0.14)	0.291

Bold values indicate statistically significant findings.

## Appendix

**Table 2:** Moderation analyses of diabetes and obesity status on cognitive function.

Variables	Animal fluency		Stroop interference		Mean reaction		Rey I (immediate)		Rey II (delayed)	
	*b* (95% CI)	*p*	*b* (95% CI)	*p*	*b* (95% CI)	*p*	*b* (95% CI)	*p*	*b* (95% CI)	*p*
**Age**	**-0.16** **(-0.16, -0.15)**	**<0.001**	**0.19** **(0.18, 0.20)**	**<0.001**	**5.76** **(5.54, 5.98)**	**<0.001**	**-0.05** **(-0.05, -0.05)**	**<0.001**	**-0.06** **(-0.06, -0.06)**	**<0.001**
Sex										
Female	Ref		Ref		Ref		Ref		Ref	
Male	0.13 (0.00, 0.26)	0.050	**0.48** **(0.32, 0.63)**	**<0.001**	**-19.55** **(-23.61, -15.50)**	**<0.001**	**-0.83** **(-0.87, -0.79)**	**<0.001**	**-1.00** **(-1.05, -0.95)**	**<0.001**
Ethnicity										
Non-white	Ref		Ref		Ref		Ref		Ref	
White	**3.40** **(3.09, 3.70)**	**<0.001**	**-1.04** **(-1.40, -0.67)**	**<0.001**	**-74.45** **(-83.06, -64.83)**	**<0.001**	**0.45** **(0.35, 0.55)**	**<0.001**	**0.50** **(0.38, 0.62)**	**<0.001**
Income										
No response	**-1.17** **(-1.49, -0.85)**	**<0.001**	0.35 (-0.03, 0.72)	0.070	**10.13** **(0.16, 20.10)**	**0.046**	**-0.26** **(-0.37, -0.16)**	**<0.001**	**-0.29** **(-0.41, -0.17)**	**<0.001**
< $20,000	**-1.88** **(-2.25, -1.52)**	**<0.001**	**1.97** **(1.53, 2.40)**	**<0.001**	**46.30** **(34.79, 57.81)**	**<0.001**	**-0.73** **(-0.85, -0.61)**	**<0.001**	**-0.44** **(-0.58, -0.30)**	**<0.001**
$20,000 to< $50,000	**-1.31** **(-1.54, -1.08)**	**<0.001**	**0.93** **(0.66, 1.20)**	**<0.001**	**22.82** **(15.64, 30.00)**	**<0.001**	**-0.37** **(-0.45, -0.29)**	**<0.001**	**-0.14** **(-0.23, -0.06)**	**0.001**
$50,000 to< $100,000	**-0.76** **(-0.94, -0.58)**	**<0.001**	0.09 (-0.12, 0.31)	0.405	**9.66** **(3.91, 15.41)**	**0.001**	**-0.14** **(-0.20, -0.08)**	**<0.001**	0.03 (-0.04, 0.10)	0.346
$100,000 to< $150,000	**-0.25** **(-0.44, -0.06)**	**0.009**	0.07 (-0.15, 0.30)	0.526	**6.54** **(0.59, 12.48)**	**0.031**	-0.06 (-0.12, 0.00)	0.067	**0.11** **(0.03, 0.18)**	**0.004**
< $150,000 or more	Ref		Ref		Ref		Ref		Ref	
Education										
High school or less	**-2.77** **(-2.98, -2.57)**	**<0.001**	**1.53** **(1.28, 1.77)**	**<0.001**	**7.78** **(1.30, 14.25)**	**0.018**	**-0.86** **(-0.93, -0.79)**	**<0.001**	**-0.90** **(-0.98, -0.82)**	**<0.001**
Below bachelor	**-1.79** **(-1.92, -1.65)**	**<0.001**	**0.73** **(0.57, 0.90)**	**<0.001**	**4.99** **(0.62, 9.36)**	**0.025**	**-0.51** **(-0.56, -0.46)**	**<0.001**	**-0.54** **(-0.59, -0.49)**	**<0.001**
Bachelor or above	Ref		Ref		Ref		Ref		Ref	
Residence										
Urban	Ref		Ref		Ref		Ref		Ref	
Rural	-0.20 (-0.42, 0.02)	0.072	0.23 (-0.03, 0.49)	0.079	**-15.78** **(-22.67, -8.89)**	**<0.001**	0.05 (-0.02, 0.13)	0.149	-0.03 (-0.11, 0.06)	0.527
Comorbidity										
None	Ref		Ref		Ref		Ref		Ref	
1-2	0.04 (-0.13, 0.22)	0.631	0.09 (-0.12, 0.30)	0.385	2.45 (-3.07, 7.97)	0.384	-0.02 (-0.07, 0.04)	0.526	0.02 (-0.05, 0.09)	0.566
> 2	**0.22** **(0.04, 0.41)**	**0.019**	0.19 (-0.03, 0.41)	0.097	5.84 (-0.05, 11.73)	0.052	0.01 (-0.05, 0.07)	0.876	0.01 (-0.07, 0.08)	0.877
Alcohol intake										
** ≤ ** 1 time per week	Ref		Ref		Ref		Ref		Ref	
2-7 times per week	**0.33** **(0.20, 0.46)**	**<0.001**	**-0.40** **(-0.55, -0.24)**	**<0.001**	-0.38 (-4.46, 3.71)	0.856	**0.09** **(0.05, 0.14)**	**<0.001**	**0.09** **(0.04, 0.14)**	**<0.001**
Physical Activity										
Seldom	**-0.55** **(-0.68, -0.41)**	**<0.001**	0.01 (-0.15, 0.17)	0.882	-0.54 (-4.74, 3.66)	0.800	**-0.06** **(-0.10, -0.01)**	**0.010**	**-0.07** **(-0.12, -0.02)**	**0.008**
Often	Ref		Ref		Ref		Ref		Ref	
BMI										
Healthy weight	Ref		Ref		Ref		Ref		Ref	
Underweight	-0.41 (-1.21, 0.39)	0.321	0.57 (-0.38, 1.52)	0.239	5.54 (-19.58, 30.67)	0.665	**-0.33** **(-0.59, -0.06)**	**0.015**	**-0.34** **(-0.65, -0.04)**	**0.027**
Overweight	**-0.25** **(-0.41, -0.09)**	**0.002**	-0.01 (-0.20, 0.18)	0.906	-2.68 (-7.66, 2.30)	0.291	**-0.06** **(-0.12, -0.01)**	**0.016**	-0.05 (-0.11, 0.01)	0.141
Obesity	**-0.21** **(-0.39, -0.03) **	**0.023**	**0.32** **(0.11, 0.54)**	**0.004**	-4.51 (-10.26, 1.24)	0.124	**-0.08** **(-0.14, -0.02)**	**0.011**	**-0.12** **(-0.19, -0.05)**	**0.001**
Diabetes										
No diabetes	Ref		Ref		Ref		Ref		Ref	
Type 2 diabetes	-0.12 (-0.83, 0.60)	0.750	**0.94** **(0.09, 1.78)**	**0.030**	-0.43 (-22.89, 22.03)	0.970	-0.01 (-0.24, 0.23)	0.942	0.02 (-0.25, 0.29)	0.882
Other type	0.02 (-0.53, 0.57)	0.948	-0.22 (-0.87, 0.43)	0.507	1.72 (-15.54, 18.98)	0.845	0.12 (-0.06, 0.31)	0.178	**0.26** **(0.05, 0.47)**	**0.016**
Interaction										
T2DM*Underweight	-8.57 (-21.05, 3.91)	0.178	**17.02** **(2.25, 31.80)**	**0.024**	19.66 (-372.31, 411.63)	0.922	-0.44 (-4.55, 3.68)	0.834	-2.66 (-7.39, 2.07)	0.270
Other diabetes* Underweight	-1.94 (-5.62, 1.74)	0.303	2.35 (-2.00, 6.71)	0.290	7.12 (-108.42, 122.66)	0.904	-0.57 (-1.79, 0.64)	0.353	**-1.60** **(-2.99, -0.20)**	**0.025**
T2DM*Overweight	0.00 (-0.83, 0.83)	0.999	-0.07 (-1.05, 0.90)	0.881	21.11 (-4.83, 47.06)	0.111	-0.14 (-0.41, 0.14)	0.330	-0.24 (-0.56, 0.07)	0.126
Other diabetes* Overweight	-0.29 (-0.96 0.37)	0.390	0.00 (-0.79, 0.79)	1.000	-2.27 (-23.12, 18.58)	0.831	-0.15 (-0.37, 0.07)	0.173	**-0.33** **(-0.58, -0.08)**	**0.010**
T2DM*Obesity	0.01 (-0.77, 0.79)	0.978	-0.57 (-1.50, 0.35)	0.226	18.12 (-6.48, 42.71)	0.149	-0.08 (-0.34, 0.18)	0.559	-0.08 (-0.38, 0.21)	0.583
Other diabetes*Obesity	0.32 (-0.33, 0.98)	0.331	0.29 (-0.48, 1.06)	0.462	5.30 (-15.24, 25.83)	0.613	-0.05 (-0.26, 0.17)	0.674	-0.15 (-0.40, 0.10)	0.229

Bold values indicate statistically significant findings.
